# Tumour pharmacodynamics and circulating cell free DNA in patients with refractory colorectal carcinoma treated with regorafenib

**DOI:** 10.1186/s12967-015-0405-4

**Published:** 2015-02-12

**Authors:** Andrea Li Ann Wong, Joline Si Jing Lim, Arvind Sinha, Anil Gopinathan, Robert Lim, Chee-Seng Tan, Thomas Soh, Sudhakar Venkatesh, Christina Titin, Nur Sabrina Sapari, Soo-Chin Lee, Wei-Peng Yong, David Shao Ping Tan, Brendan Pang, Ting-Ting Wang, Ying-Kiat Zee, Richie Soong, Zuzana Trnkova, Chetan Lathia, Jean-Paul Thiery, Scott Wilhelm, Michael Jeffers, Boon-Cher Goh

**Affiliations:** Department of Haematology-Oncology, National University Health System, 1E Kent Ridge Road, Singapore, 119228 Singapore; Cancer Science Institute, Centre for Translational Medicine, 14 Medical Drive, #12-01, Singapore, 117599 Singapore; Department of Diagnostic Imaging, National University Health System, 1E Kent Ridge Road, Singapore, 119228 Singapore; Department of Pathology, National University Health System, 1E Kent Ridge Road, Singapore, 119228 Singapore; Bayer Healthcare Pharmaceuticals, 100 Bayer Boulevard, PO Box 915, Whippany, NJ 07981-0915 USA

**Keywords:** Regorafenib, Colorectal carcinoma, Pharmacodynamics, Plasma cell-free DNA

## Abstract

**Background:**

Regorafenib, a multi-kinase inhibitor, is used in the treatment of patients with metastatic colorectal cancer refractory to standard therapy. However, this benefit was limited to 1.4 months improvement in overall survival, with more than half of patients experiencing grade 3 to 4 adverse events. We aim to elucidate the pharmacodynamic effects of regorafenib in metastatic colorectal cancer and discover potential biomarkers that may predict clinical benefit.

**Methods:**

Patients with metastatic colorectal adenocarcinoma refractory to standard therapy with tumours amenable to biopsy were eligible for the study. Regorafenib was administered orally at 160 mg daily for 3 out of 4 weeks with tumour assessment every 2 cycles. Metabolic response was assessed by FDG PET-CT scans (pre-treatment and day 15); paired tumour biopsies (pre-treatment and day 21 post-treatment) were sampled for immunohistochemistry and proteomic profiling analyses. Plasma circulating cell free DNA was quantified serially before and after treatment.

**Results:**

There were 2(6%) partial responses out of 35 patients, and 8(23%) patients had stable disease for more than 7 months. Adverse event profile was similar to reported data. Recurrent somatic mutations in *K-RAS*, *PIK3CA* and *BRAF* were detected in plasma circulating cell free DNA in 14 patients; some mutations were not found in archival tumour. Total plasma circulating cell free DNA inversely correlated with progression free survival (PFS), and presence of KRAS mutations associated with shorter PFS. Immunohistochemistry of pre- and post- treatment biopsies showed majority of patients had downregulation of phosphorylated-VEGFR2, podoplanin, phosphorylated-AKT, Ki-67 and upregulation of the MEK-ERK axis, phosphorylated-C-MET, phosphorylated-SRC, phosphorylated-STAT3 and phosphorylated-JUN. Proteomic analysis of fine needle tumour aspirates showed down-regulation of PI3K was associated with longer PFS.

**Conclusion:**

Plasma circulating cell free DNA may yield potential predictive biomarkers of regorafenib treatment. Downregulation of the PI3K-AKT axis may be an important predictor of clinical benefit.

**Electronic supplementary material:**

The online version of this article (doi:10.1186/s12967-015-0405-4) contains supplementary material, which is available to authorized users.

## Background

Regorafenib is a multi-kinase inhibitor with activity in nanomolar concentration range against VEGFR1-3, PDGFR, C-KIT, RET, TIE-2, C-RAF, B-RAF, p38-αand FGFR1. [1] A randomised phase III study of regorafenib against placebo and best supportive care in patients with refractory colorectal carcinoma (CRC) including prior therapy with bevacizumab, cetuximab or panitumumab in *KRAS* wild-type tumours showed improvement of progression free survival (PFS) as well as overall survival (OS) in favour of regorafenib treatment, establishing a new standard of care in this study population [2].However, OS benefit was a modest 1.4 months, contributed mainly by disease control rather than tumour shrinkage. Analysis of PFS and OS plots clearly suggest a subpopulation of patients with refractory CRC that would benefit from regorafenib therapy. Regorafenib therapy carries potential risk of adverse events including hepatotoxicity, haemorrhage, hand-foot syndrome, coronary syndromes, and reversible posterior leukoencephalopathy syndrome. In the pivotal phase III study, 61% of patients had dose interruption, 38% had dose reduction, and 8.2% discontinued therapy due to adverse events. Therefore, better selection of patients through elucidation of the mechanism of action of regorafenib and development of biomarkers to predict clinical benefit is critical. We designed an open label study of regorafenib in Asian patients with metastatic refractory CRC to determine the molecular underpinnings of regorafenib treatment as well as to develop biomarkers that could potentially predict clinical benefit.

## Methods

### Patient eligibility

Eligible patients had histologically verified, biopsy amenable metastatic colorectal adenocarcinoma refractory to standard therapy and not amenable to surgery with curative intent. Biopsy amenable was defined as ≥1 lesion suitable for repeated biopsy; *e.g.* subcutaneous nodule, skin lesion, rectal tumour, colonic mass easily reached by colonoscopy, peritoneal masses ≥3 cm in maximum diameter easily assessable by image guided core biopsy, or liver lesions ≥3 cm in maximum dimension with a rim of normal liver tissue, accessible safely by image guided core biopsy using an 18 F gauge needle as determined by an experienced interventional radiologist. Other criteria included ECOG (Eastern Cooperative Oncology Group) Performance Score of 0–1, bone marrow function, liver function and renal function within normal limits, and life expectancy of ≥3 months. Patients were excluded if they had undergone major surgery, chemotherapy, investigational therapy or radiotherapy within 28 days of start of regorafenib, severe illnesses or malabsorption. All patients gave written informed consent and the study was approved by the Domain Specific Ethics Review Board of the National Healthcare Group, Singapore and the Health Sciences Authority of Singapore (Clinicaltrials.gov identifier NCT1189903).

## Materials and methods

The study was an open-label study conducted at the National University Cancer Institute, Singapore. Regorafenib tablets (40 mg) were supplied by Bayer Healthcare Berlin, Germany and administered orally after breakfast at a starting dose of 160 mg daily for 21 days followed by 7 days without dosing per 28-day cycle. Dosing was continued until disease progression, occurrence of unacceptable toxicity, withdrawal of consent or withdrawal at physician’s discretion. Plasma samples were collected on cycle 1 day 1, cycle 1 day 8 and cycle 2 day 21 prior to dosing for circulating cell free DNA analysis. Tumour biopsies were performed using 18G core biopsy needles limited to 1–2 passes for each sample to reduce risk of haemorrhage and fine needle aspirations using 23G needles with immediate bedside cytopathological confirmation of presence of tumour cells; biopsies were done within 1 week of initiating regorafenib, and repeated on day 21. Tumour biopsies were processed according to procedures detailed below. Immunohistochemistry (IHC) analysis of tumour samples were performed at Mosaic Laboratories (Lake Forest, California, USA). Fine needle aspirates were stored in Protein Later® in glass cryovials and shipped to Prometheus Laboratories (San Diego, California, USA). Proteomic analysis and quantitation from aspirates were performed by Collaborative Enzyme Enhanced Reactive Immunoassay (CEER).

Dose interruptions were permitted for adverse events of grade 2 or higher; up to 2 dose reductions were allowed in each patient for toxicities of grade 3 or 4.

### Tissue microscopy and IHC on formalin fixed, paraffin embedded tissues

Fifty-five samples were stained with H&E, and stained slides were evaluated by a pathologist for presence and percentage of tumour and necrosis. IHC was performed in accordance with Mosaic Laboratories’ standard operating procedures (SOP).

Stained slides were reviewed by image analysis (ImageScoop software, Aperio, Vista CA) for CD31, podoplanin, and Ki-67. The final output for CD31 and Ki-67 was total percent positive staining using Aperio algorithms. The final output for podoplanin was a hotspot count of three areas of lymphatic vessels. Areas with the highest amount of lymphatic vessels were identified and three fields at 20x magnification were selected for manual enumeration.

All other stains were reviewed by a pathologist blinded to patient treatment. Pathology review consisted of a semi-quantitative evaluation of staining in the tumour at four levels: 0 (unstained), 1+ (weak staining), 2+ (moderate staining) and 3+ (strong staining). Percent positive staining and H-Score were calculated based on the summation of the product of percent of cells stained at each intensity using the following equation: (3 × % cells staining at 3+) + (2 × % cells staining at 2+) + (1 × % cells staining at 1+). Both nuclear and cytoplasmic staining were factored into the H score.

IHC stains used included phosphorylated VEGFR2 (pVEGFR2), total VEGFR2, phosphorylated ERK (pERK), total ERK, CD31, phosphorylated c-Kit (pKIT), total c-Kit, phosphorylated MEK (pMEK), phosphorylated JUN (pJUN), phosphorylated JNK (pJNK), podoplanin, phosphorylated STAT3 (pSTAT3), total STAT3, phosphorylated AKT (pAKT), total AKT, phosphorylated MET (pMET), cMET, HGF, phosphorylated p70 S6 kinase Thr389 (pp70S6K), phosphorylated SRC (pSRC), and total SRC. All IHC assays have been optimized and validated for use in human tissue. All tissues were screened by H&E and a subset of suitable samples were screened by IHC.

Details of IHC antibodies are elaborated in Additional 1.

### Analysis of plasma circulating cell free DNA

Fourteen somatic mutations were analysed in DNA isolated from plasma samples obtained on cycle 1 day 1 prior to treatment, cycle 1 day 8, and cycle 2 day 21. The list of mutations studied were part of a panel of genes specific for colorectal cancer (Additional file 2: Table S1). Analysis was done using BEAMing (Beads, Emulsions, Amplification and Magnetics) technology at InosticsGmBH, Hamburg, Germany [3]. Plasma samples are first thawed at room temperature before DNA preparation, with cell debris removed by centrifugation. The QIAamp CAN purification kit was then used for isolation of free circulating DNA from plasma samples. Quantification was done using a modified version of human LINE-1 qRT-PCR. Human LINE-1 sequence-based assay, rather than individual genes, was chosen to measure DNA yield after DNA isolation as it required a smaller amount of starting material. Each sample and reference standard were run in duplicate. Isolated plasma DNA was then pre-amplificed under high-fidelity PCR conditions, and PCR products were quality checked on an agarose gel. The resultant pre-amplified DNA was then utilised for subsequent BEAMing assay. Normalized amounts of pre-amplified DNA was then further amplified on magnetic bead surfaces in water in oil-emulsions, and hybridized to fluorescent labelled mutation specific probes and subjected to flow cytometry to quantify the fraction of mutant to wild-type DNA alleles. This fraction is calculated by dividing the amount of mutant beads by total amount of beads with PCR product, with lower detection limit at mutant fraction of 0.02%. Total circulating cell free DNA included non-mutant and mutant DNA from tumour as well as non-mutant, non-tumour derived DNA. Quantification of DNA content was expressed as genome equivalents (GE), where 1 GE is one haploid genome weighing 3.3 pg.

### Collaborative enzyme enhanced reactivity (CEER) analysis

Tumour fine needle aspirates (FNAs) obtained with 23G gauge needle were placed in ProteinLater® solution and shipped to Prometheus Laboratories (Prometheus Laboratories, San Diego, CA) for further processing and analysis as described previously [4]. Protein lysates were prepared according to a SOP which included tissue homogenization and collection of supernatants following centrifugation at 16,000 rpm for 15 minutes at 4°C. Protein concentrations were determined using bicinchoninic acid (BCA) assay and protein lysates were aliquoted and stored at −70°C prior to CEER analysis. CEER is a proximity-based immunoassay suitable for analysing both total protein expression levels and activation status of protein signalling cascades of interest.

### Tumour imaging

Patients underwent fluorodeoxyglucose (FDG) PET-CT scans within a week before starting regorafenib and on day 15 of cycle 1. Tumour standardized uptake values (SUV) were measured according to standardized image acquisition protocols and assessed according to EORTC criteria for response. Tumour measurements were made according to RECIST 1.1 and compared every 2 cycles.

### Statistical analysis

This was an exploratory study and sample size was based on obtaining 12 to 14 paired samples suitable for IHC with an expected attrition rate of 60%. Descriptive statistics were used to present data on each biomarker in the study. PFS was defined as the time from starting treatment to clinical or radiological progression or death, whichever occurred first. Correlation between variables was performed using Pearson correlation coefficient analysis. To study the effect of change in each protein biomarker after treatment on PFS, comparison of PFS between patients with up- or down-regulated biomarkers was done using non-parametric Mann Whitney U test. The log-rank test was used to compare PFS distributions for KRAS mutation status or changes in biomarker levels after regorafenib treatment. All statistical analyses were done using IBM SPSS Statistics version 21.0.

## Results

### Drug efficacy

**Figure 1:**
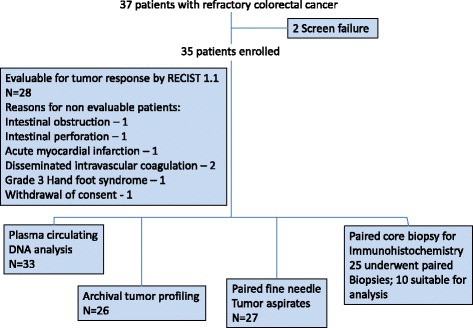
**37 patients with refractory colorectal cancer.**

Thirty-seven patients were enrolled; 2 failed screening and 35 received at least one dose of regorafenib. As expected, in this population of refractory CRC patients, 49% had 4 or more prior chemotherapy regimens and 43% had received prior bevacizumab and/or cetuximab treatment (Table 1). Figure 1 shows the CONSORT diagram of patients’ treatment, tumour response evaluation and tumour biopsies obtained. Twenty-eight patients were evaluable for response, with best response of 2 partial responses (PR) and 18 patients with stable disease (SD), resulting in a disease control rate (DCR) at 8 weeks of 57% by intent-to-treat analysis. Median PFS was 105 days (range 17–484 days); 8(23%) patients remained progression-free for 217 days or longer. Patients had similar PFS regardless of prior treatment with bevacizumab (log rank test p = 0.758).

**Table 1:** **Patient demographics and clinical characteristics**

**Parameter**	**No.**
Sex	
Male	22
Female	15
Median age, years	58
Range	27-77
ECOG performance status	
0	20
1	17
Tumor biopsy sites	
Liver	15
Abdominal wall	9
Rectum/vaginal wall	4
Scalp	2
Lymph node	5
Prior lines of treatment	
2-3	18 (51%)
≥4	17 (49%)
Prior treatment	
Bevacizumab	15 (43%)
Cetuximab	15 (43%)
Baseline organ function	
AST (U/L)	34.6 ± 22.4
ALT (U/L)	23.5 ± 10.9
Total bilirubin (μmol/L)	10.1 ± 5.8
Creatinine (μmol/L)	66.9 ± 27.0

37 patients screened; 35 commenced therapy, 2 failed screening.

8 patients did not complete cycle 2 for tumour biopsy and evaluation of response.

11 patients did not have second biopsy.

Twenty-nine patients had paired FDG PET-CT scans, and 15 (52%) patients had partial or complete metabolic response on cycle 1 day 15 according to EORTC criteria, although there was no correlation between day 15FDG PET SUV response and DCR (p = 1.0) or PFS (2-tailed p = 0.24).

### Adverse events

The toxicity profile of regorafenib was consistent with previous reports, commonest included fatigue, hand-foot syndrome (HFS), diarrhea, and hypophonation occurring in more than 30% of patients. Three deaths were considered treatment-related, all occurring in cycle 1, including acute myocardial infarction, disseminated intravascular coagulation and bowel perforation. Ten other patients developed toxicities in cycle 1 requiring dose reduction or discontinuation of treatment. There were no biopsy related complications. Median relative dose intensity was 79%, with 43% of patients requiring dose reduction, and 60% of patients requiring at least one dose interruption.

### Plasma circulating cell free DNA

Fourteen patients had quantifiable plasma circulating tumour mutant DNA on cycle 1 day 1 prior to treatment, and underwent quantification of total (derived from tumour and non-tumour tissues) as well as mutant DNA copies (from tumour tissues) on cycle 1 day 8 and cycle 2 day 21. Mutations detected included *KRAS* mutations (n = 14, 40%), *PIK3CA* mutations (n = 4, 11%) and *BRAF* (n = 3, 9%), consistent with reported frequencies. Percentage of mutant *KRAS* DNA ranged from 0.03% to 54%. Four patients had mutations that were not found in available archival tumour tissue (archival tissue available for 26 patients); 3 were *PIK3CA* mutations, and 1 had multiple mutations in *BRAF*, *KRAS* and *NRAS*. These could reflect tumour heterogeneity, genetic evolution of the tumour with treatment, or both. Two patients who received prior cetuximab treatment (patient 19 and 35) did not have detectable *KRAS* mutations in archival tissue, but had multiple KRAS mutations in plasma cell free DNA occurring in low frequency.

**Figure 2:**
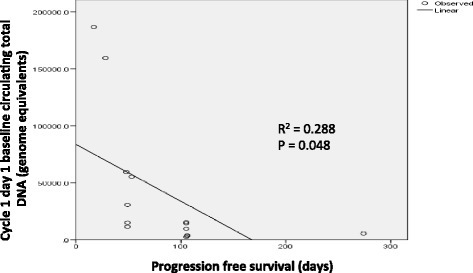
**Correlation between PFS and baseline total cell free DNA.**

Total circulating cell free DNA at baseline inversely correlated with PFS; r = 0.537, p = 0.048 (Figure 2). Total plasma DNA remained lower in patients with longer PFS even after regorafenib treatment (Additional file 3: Figure S1). Baseline mutant DNA copy number and percentage (%) mutant DNA were not predictive of PFS; r = −0.38, p = 0.18 and r = −0.27, p = 0.35, respectively.

Patients with detectable *KRAS* mutations in circulating cell free DNA had shorter PFS compared to patients with undetectable *KRAS* mutations (mean PFS 105 days [95% CI 58–152 days] vs 217 days [95% CI 76–358 days] p = 0.04). Additional file 3: Figure S2 shows trends of % mutant DNA with time in patients with PFS below compared to above median PFS. All except 1 patient (patient 13) with longer PFS had early decline in mutant DNA fraction and remained at a low level, whereas patients with shorter PFS had increased or little change in mutant DNA fraction at day 8. Patients 19, 26 and 33 in the latter group had fall in mutant DNA fraction on day 8, and all had metabolic response on PET CT on day 15.

### Immunohistochemistry changes in tumour before and after regorafenib

**Figure 3:**
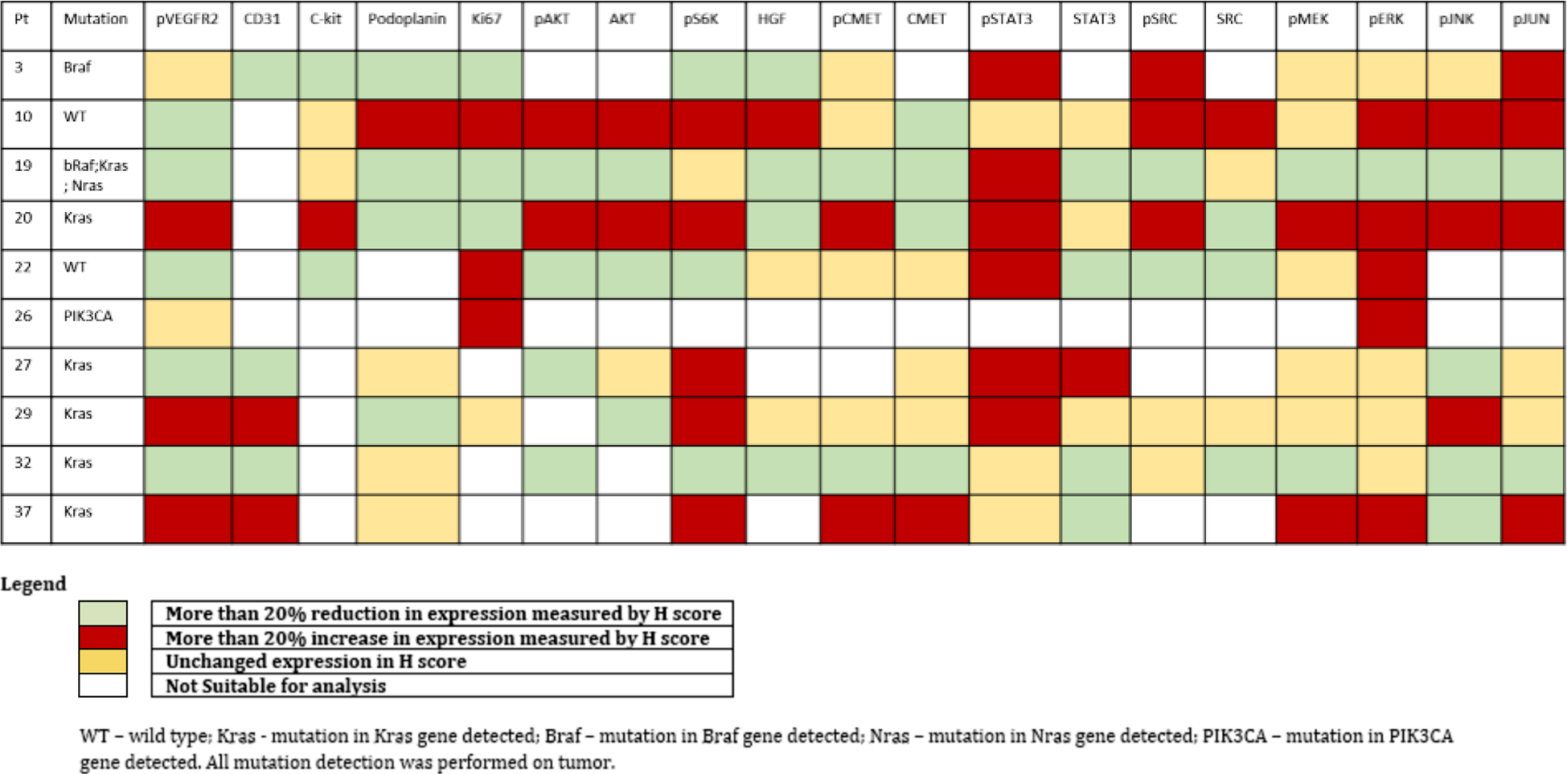
**Changes in immunohistochemistry with regorafenibtreatment.**

**Figure 4:**
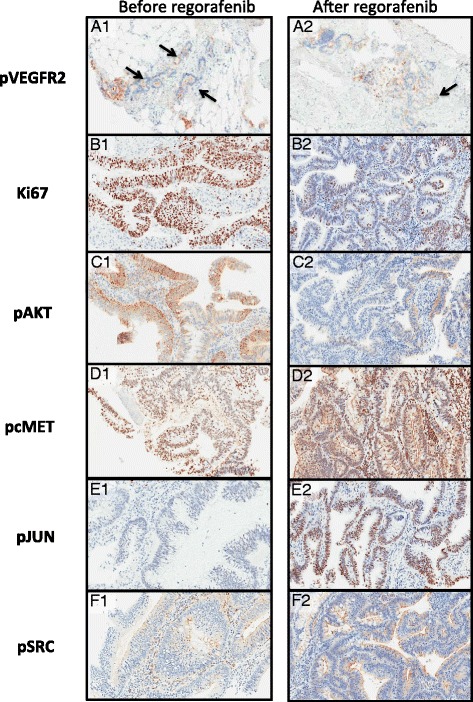
**Immunohistochemistry of selected proteins of representative samples before and after regorafenib treatment.** Patient 27: pVEGFR2 staining shoes a reduced H score of pre-trearment-35 (A1: 5% 2+; 30% 1 + staining) to post-treatment-20 (A2: 5% 2+; 15% 1+ staining) in a small proportion of tumor cells. Arrows denote reduced proportion of tumor cells with 1+ staining between pre and post-treatment samples. Patient 20: Ki67 staining shows reduced expression of pre-treatment (B1: 63%) to post-treatment (B2: 26%); pAKT staining shows a reduced H score of pre-treatment-10 (C1: 6% 3+; 2% 2+; 2% 1+; 90% 0) to post-treatment (C2: 2% 3+; 8% 2+; 10% 1+; 80% 0); pcMET staining shows an increased H score of pre-treatment-80 (D1: 0% 3+; 2%+; 78% 1+; 20% 0) to post-treatment -95 (D2: 20% 3+; 5% 2+; 70% 1 + 5% 0); pJUN staining shows an increased H score of pre-treatment-5 (E1: 0% 3+; 0% 2+; 5% 1+; 95% 0) to post-treatmetn-50 (E2: 2% 3+; 13% 2+; 35% 1+; 50% 0); pSRC staining shows an increased H score of pre-treatmetn-3 (F1: )% 3+; 0% 2+; 3% 1+; 97% 0) to post-treatment-35 (F2: 15% 3+; 10% 2+; 10% 1+; 65% 0). Only staining in tumor cells was scored, background and stromal staining was ignored.

After assessing the quality of tissue obtained and representativeness of tumour samples on H&E stains, 10 out of 25 paired samples were suitable for analysis for tumour effects of regorafenib treatment. Figure 3 summarises changes in H-scores in post-treatment samples compared to baseline. pVEGFR-2, podoplanin, CD31, Ki67, pKIT, pAKT, c-MET, STAT3 and HGF were downregulated whereas pMET, pMEK, pSRC, pSTAT3 and pJUN were upregulated in majority of patients. Selected IHC results for patients 20 and 27 are illustrated in Figure 4, reflecting increased and reduced expression for pVEGFR-2, Ki67, pAKT, pc-MET, pJUN and pSRC.

### Proteomic analysis of tumour aspirates

**Figure 5:**
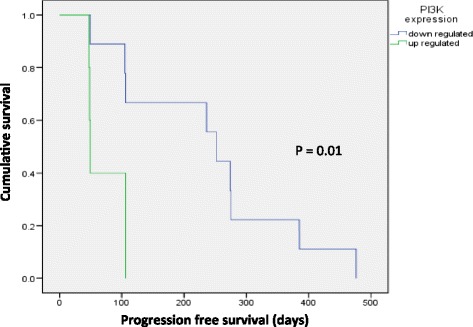
**Progression free survival of patients according to change in PI3K expression.**

Tumour aspirates preserved in ProteinLater® were analysed on a printed array panel format, with each immunoassay normalised to cytokeratin expression and measured in computed units (CU). We chose proteins available on the panel and considered relevant to molecular targets of regorafenib or commonly activated signalling pathways in colorectal cancer. Fourteen paired samples were analysed, and changes in expression were compared before and after treatment. To evaluate predictors for PFS, log-rank analyses of PFS between patients with up- or down-regulation (defined as ≥20% increase or decrease in CU score post treatment) of each protein co-variable were performed. Down regulation of PI3K was observed in 10/14 (71%) of patients and associated with prolonged PFS (median PFS 252 days in down regulated vs 49 days in unregulated patients; log rank p = 0.01) (Figure 5). Table 2 shows percentage change in various proteins in the tumour aspirate after treatment with regorafenib. We correlated changes in protein expression according to metabolic response, and found that down-regulation of the phosphorylated-proline rich AKT substrate (pPRAS) was seen in all patients with metabolic response (n = 8), compared to 25% of patients without metabolic response (n = 4, Fishers exact test p = 0.018).

**Table 2:** **Changes in protein expression of various biomarkers and their mutational status and progression free survival**

	**Mutational status of tumor**	**PFS (Days)**	**PI3K (% change)**	**pPI3K (% change)**	**IGF1R (% change)**	**pMEK (% change)**	**cMET (% change)**	**pERK (% change)**	**pAKT (% change)**
	*BRAF*	385	-99.5	-97.1	-93.6	-	-72.3	-	-83.9
	Wt	275	-46.3	-75.3	-61	-92.9	-75.8	-96.8	-90.4
	*KRAS* + *PIK3CA*	274	-39.7	54.9	0	0	378.7	1570	2653.9
	Wt	252	-97.7	0	-59.3	0	-86	-93.3	-61.5
	Wt	484	-86	-86.8	-95.3	0	-97.1	-87.5	210
	Wt	236	-63.2	-96.7	-99.4	-94.2	-73.1	-98.1	-98.7
	Wt	109	-99.8	0	59.1	0	1261	30.6	-64.3
	*BRAF*	106	483.3	97.3	1630	0	473.3	540	975
	*NRAS*	106	-92.7	-96.8	-97.2	-92.5	-93.5	-99	-64.3
	*KRAS* + *PIK3CA*	105	-99.9	0	0	0	1864.5	0	-81.8
	*KRAS*	48	36020	-38.3	38.6	730	561.7	1490	2653.9
	*KRAS*	47	180	770	7.6	-37.1	324.8	1.3	571
	*BRAF* + *KRAS*	49	159.7	-96.5	12.7	-94.2	20	-81.6	-97.5
	*PIK3CA* + *NRAS*	49	-85.7	-99.6	-99.9	-97.6	-86.4	-96.2	-81.3
*Test of null hypothesis P value			0.01	0.50	0.12	0.57	0.07	0.53	0.70

Wt – Wild-type.

Negative % change reflects downregulation; positive % change reflects upregulation.

*Mann Whitney U test (2-tailed) of PFS between patient populations with upregulated or downregulated protein expression.

## Discussion

Regorafenib had similar efficacy and toxicity profile in this study as presented in the CORRECT study [2]. Treament related mortalities were observed and dose reduction for toxicities was common in the first cycle. Nonetheless, as a substantial proportion of patients experienced significant clinical benefit, it is critical to characterise this subgroup. Compared to the CORRECT study where all patients had prior bevacizumab, 43% of patients had prior bevacizumab in this study; clinical benefit was similar with or without bevacizumab. This study provided some biomarker leads regarding patients who have longer PFS and a potential mechanism of resistance to regorafenib through the PI3K-AKT pathway of signalling.

Circulating cell free DNA analysis identified mutant *KRAS*, *PIK3CA* and *BRAF* with frequencies consistent with reported literature and wide interindividual variability of mutant fractions [5-7]. New mutations in *RAS* and *PIK3CA* emerged after therapy compared to archival tumour, and the emergence of multiple *KRAS* mutations in low levels after anti-EGFR therapy is a known resistance mechanism to anti-EGFR antibodies [8]. These findings raise the possibility of evaluating mechanism-based treatments like MEK inhibitors and PI3K inhibitors in these patients with cetuximab resistance according to detection of *KRAS* or *PIK3CA* mutations in plasma cell free DNA, respectively.

There has been controversy regarding the use of cell free DNA fractions as predictive biomarkers; both total circulating DNA and mutant fractions have been correlated with tumour response. Consistent with a previous report, we found that total circulating cell free DNA levels correlated inversely with PFS Total plasma cell free DNA includes DNA from both mutant and non-mutant tumour cells, and non-tumour cells, and thus may be a better representation of the overall tumour burden in a patient with metastatic cancer where variability of mutant fraction exists due to tumour heterogeneity. Our data and findings from renal cell carcinoma support this finding [9,10]. Furthermore, as tumour subclones vary in sensitivity to treatment, circulating mutant DNA derived from tumour subclones may not be an accurate reflection of overall tumour burden. On the other hand, quantitation of circulating mutant DNA showed early decrease in the mutant fraction in patients with longer PFS, suggesting that in colorectal carcinoma, early reduction in the more aggressive mutant clones could prolong disease control. This observation is consistent with previous studies in CRC patients undergoing surgery where complete resections resulted in a sharp drop in circulating mutant DNA 24 hours after surgery, and in breast cancer patients where a reduction in mutant DNA tumour fraction correlated with early tumour response to treatment [11].

Immunohistochemistry of paired tumour samples showed that regorafenib targets tumour stromal vasculature through down regulation of VEGFR-2 phosphorylation and podoplanin. However, several signalling pathways including expected targets of regorafenib were upregulated, perhaps suggesting alternative pathways were activated in response to regorafenib [12]. For example, the up regulation of pMEK and lack of suppression of ERK phosphorylation was surprising given both VEGFR-2 and RAF are inhibited by regorafenib. Nonetheless, this is consistent with *in vitro* studies that show early decrease in pERK upon initial exposure, followed by increase upon longer exposure to regorafenib with increased MEK phosphorylation [13]. Only the AKT pathway appeared to be downregulated consistently from regorafenib treatment. In cell lines, regorafenib has been shown to induce endoplasmic reticulum stress, and the observation that regorafenib treatment activated JUN in most patients is consistent with preclinical studies of regorafenib in hepatocellular cell lines where treatment with regorafenib resulted in upregulation of pJNK [13,14].

CEER analysis indicate the importance of the PI3K-AKT-mTOR axis in mediating tumour survival mechanisms, as PI3K down regulation in 71% of patients and was associated with longer PFS and metabolic responses were more common in patients with downregulation of pPRAS. The patient samples studied were limited, however, these findings should warrant a larger study to validate the biomarkers. If so, biomarkers in this pathway could inform the design of studies selecting patients for combination treatment with regorafenib and PI3K-AKT-mTOR inhibitors. It has been shown that regorafenib has synergistic antitumor effects with inhibitors of PI3K/AKT pathway, particularly against colorectal carcinoma HCT116 both *in vitro* and *in vivo* [15]. Taken together with our data, this suggests a role for combination of regorafenib with either a PI3K or AKT inhibitor in patients whose tumours activate PI3K-AKT following regorafenib treatment. We have initiated preclinical studies to understand mechanistically the role of inhibitors of the PI3K-AKT-mTOR axis in relation to regorafenib treatment in colorectal carcinoma cell lines, and if promising, will proceed to a clinical trial of addition of a PI3K or AKT inhibitor in patients with metastatic colorectal carcinoma who have demonstrated refractoriness to regorafenib, selected on the basis of up regulation of the PI3K/AKT axis.

## Conclusion

In conclusion, this study raises several important hypotheses including whether circulating cell free DNA information could be utilised to monitor patients’ response to regorafenib therapy, and to guide additional treatment directed against emergence of new oncogenic mutations, as well as the role of the PI3K-AKT pathway in resistance to regorafenib. We believe that these questions should be the subject of future clinical trials.

## Additional files

Additional file 1:
**Immunohistochemistry methods - Mosaic Laboratories Test Articles.**
Additional file 2:
**List of mutations studied in plasma BEAMing analysis.**
Additional file 3: Figure S1. Time course of total plasma cell free DNA. **Figure S2.** Trend of % mutant cell free DNA with treatment time.

## Abbreviations

BCA: Bicinchoninic acid
CEER: Collaborative enzyme enhanced reactive immunoassay
Cf-DNA: Circulating cell-free DNA
CRC: Refractory colorectal carcinoma
CU: Computed units
DCR: Disease control rate
ECOG: Eastern cooperative oncology group
FDG: Fluorodeoxyglucose
FNAs: Fine needle aspirates
GE: Genome equivalents
HFS: Hand-foot syndrome
OS: Overall survival
PFS: Progression free survival
pp70S6K: Phosphorylated P70 S6 Kinase Thr389
PR: Partial responses
SOPs: Standard operating procedures
SUV: Standardized uptake values
