# Supplementary material for: Tumour pharmacodynamics and circulating cell free DNA in patients with refractory colorectal carcinoma treated with regorafenib
Source: J Transl Med. 2015 Feb 12;13:57. doi: 10.1186/s12967-015-0405-4 (PMC4332724; doi:10.1186/s12967-015-0405-4)
Supplement: Additional file 1: — Immunohistochemistry methods - Mosaic Laboratories Test Articles. [file 12967_2015_405_MOESM1_ESM.docx]

**Supplementary Methods**

**Immunohistochemistry methods - Mosaic Laboratories Test Articles**

The pVEGFR2 rabbit clone 15D2 antibody (Catalog# 4991, Lot# 3, Expiration Date Jul2013) was purchased from Cell Signaling (Danvers, MA) and stored at -20°C.

The total VEGFR2 rabbit clone 55B11 antibody (Catalog# 2479, Lot# 18, Expiration Date Oct2013) was purchased from Cell Signaling and stored at -20°C.

The pERK rabbit clone 20G11 antibody (Catalog# 4376, Lot# 10, Expiration Date May2013 and Lot # 16, expiration Date May 2014) was purchased from Cell Signaling and stored at -20°C.

The total ERK mouse clone 3A7 antibody (Catalog# 9107, Lot# 7, Expiration Date May2013) was purchased from Cell Signaling and stored at -20°C.

The CD31 mouse clone JC70A antibody (Catalog# M0823, Lot# 54859, Expiration Date Jul2012 and Lot #79268, Expiration Date Dec2014) was purchased from Dako (Carpinteria, CA) and stored at 2-8°C.

The CD34 mouse clone QBEnd/10 antibody (Catalog# CM084C, Lot# 070111, Expiration Date Jul2014) was purchased from Biocare Medical (Concord, CA) and stored at 2-8°C.

The pKIT rabbit polyclonal antibody (Catalog# 3391, Lot# 10, Expiration Date Aug2013 and Lot # 12,

Expiration Jul2014) was purchased from Cell Signaling and stored at -20°C.

The total c-KIT mouse clone 2E4 antibody (Catalog# 18-0384, Lot# 1011898A, Expiration Date Jan2013) was purchased from Invitrogen (Carlsbad, CA) and stored at 2-8°C.

The pMEK rabbit polyclonal antibody (Catalog# 9121, Lot# 31, Expiration Date Oct2012 and Aug2014) was purchased from Cell Signaling and stored at -20°C.

The pJUN rabbit clone D47G9 antibody (Catalog# 3270, Lot# 5, Expiration Date Oct2014) was purchased from Cell Signaling and stored at -20°C.

The pJNK rabbit clone 81E11 antibody (Catalog# 4668, Lot# 9, Expiration Date Oct2014) was purchased from Cell Signaling and stored at -20°C.

The podoplanin mouse clone D2-40 antibody (Catalog# M3619, Lot# 10069123, Expiration Date

Aug 2014) from Dako and stored at 2-8°C.

The Ki-67 mouse clone MiB-1 antibody (Catalog# M7240, Lot# 75880, Expiration Date Aug 2014) was

purchased from Dako and stored at 2-8°C.

The pSTAT3 Tyr705 (D3A7) rabbit polyclonal antibody (Catalog# 9145, Lot#22, Expiration Date Apr2014) was purchased from Cell Signaling and stored at -20°C.

The total STAT3 mouse clone 124H6 antibody (Catalog# 9139, Lot# 7, Expiration Date Feb2015) was

purchased from Cell Signaling and stored at -20°C.

The pAKT rabbit clone 14-5 antibody (Catalog# M3628, Lot# 10073972, Expiration Date Jan2014) was

purchased from Dako and stored at 2-8°C.

The total AKT rabbit clone C67E7 antibody (Catalog# 4691, Lot# 11, Expiration Date Nov2014) was

purchased from Cell Signaling and stored at -20°C.

The pMET rabbit polyclonal antibody (Catalog# 44888G, Lot# 985503A, Expiration Date Mar2014) was purchased from Invitrogen and stored at -20°C.

The cMET rabbit clone SP44 antibody (Catalog# M3440, Lot# 121029, Expiration Date Oct2015) was

purchased from Spring Biosciences (Pleasanton, CA) and stored at 2-8°C.

The HGF goat polyclonal antibody (Catalog # AF-294-NA, Lot# ALP0211081, Expiration Date Jul2013)

was purchased from R&D Systems (Minneapolis, MN) and stored at -20°C.

The pSRC rabbit polyclonal antibody (Catalog# 2101, Lot# 18, Expiration Date Jan2014) was purchased from Cell Signaling and stored at -20°C.

The total SRC rabbit clone 36D10 antibody (Catalog# 2109, Lot# 4, Expiration Date Jan2014) was

purchased from Cell Signaling and stored at -20°C.

The mouse IgG isotype control antibody (Lot# 15882, Expiration Date Apr2018 and Lot# 38671,

Expiration Date Jul2019) was purchased from BD Pharmingen (San Diego, CA) and stored at 2-8°C.

The rabbit IgG isotype control antibody (Lot# 51308, Expiration Date Mar2015 and Lot# 73343, Expiration Date May2017) was purchased from Dako and stored at 2-8°C.

The goat IgG isotype control antibody (Lot# 105430, Expiration 29Jan2014) was purchased from Jackson Immuno Research (West Grove, PA) and stored at 2-8°C.

**Plasma cell free DNA**

**Table 1. List of mutations studied in plasma BEAMing analysis**

| Gene | Exon | Nucleotide | | | Amino acid change | Sensitivity  (Plasma) |
| --- | --- | --- | --- | --- | --- | --- |
|  |  | Position | Change | Report |  |  |
| BRAF | 15 | 1799 | T>A | t1799a | V600E | 0.02% |
| KRAS | 1 | 34 | G>A | g34a | G12S | 0.02% |
| KRAS | 1 | 34 | G>T | g34t | G12C | 0.02% |
| KRAS | 1 | 34 | G>C | g34c | G12R | 0.02% |
| KRAS | 1 | 35 | G>A | g35a | G12D | 0.02% |
| KRAS | 1 | 35 | G>C | g35c | G12A | 0.02% |
| KRAS | 1 | 35 | G>T | g35t | G12V | 0.02% |
| KRAS | 1 | 38 | G>A | g38a | G13D | 0.02% |
| KRAS | 2 | 183 | A>C | a183c | Q61H | 0.02% |
| KRAS | 3 | 436 | G>A | g436a | A146T | 0.02% |
| NRAS | 3 | 181 | C>A | c181a | Q61K | 0.02% |
| NRAS | 3 | 182 | A>T | a182t | Q61L | 0.02% |
| PIK3CA | 9 | 1624 | G>A | g1624a | E542K | 0.02% |
| PIK3CA | 20 | 3140 | A>G | a3140g | H1047R | 0.02% |
