# Supplementary material for: Tumour pharmacodynamics and circulating cell free DNA in patients with refractory colorectal carcinoma treated with regorafenib
Source: J Transl Med. 2015 Feb 12;13:57. doi: 10.1186/s12967-015-0405-4 (PMC4332724; doi:10.1186/s12967-015-0405-4)
Supplement: Additional file 2: — List of mutations studied in plasma BEAMing analysis. [file 12967_2015_405_MOESM2_ESM.docx]

| Gene | Exon | Nucleotide | | | Amino acid change | Sensitivity  (Plasma) |
| --- | --- | --- | --- | --- | --- | --- |
|  |  | Position | Change | Report |  |  |
| BRAF | 15 | 1799 | T>A | t1799a | V600E | 0.02% |
| KRAS | 1 | 34 | G>A | g34a | G12S | 0.02% |
| KRAS | 1 | 34 | G>T | g34t | G12C | 0.02% |
| KRAS | 1 | 34 | G>C | g34c | G12R | 0.02% |
| KRAS | 1 | 35 | G>A | g35a | G12D | 0.02% |
| KRAS | 1 | 35 | G>C | g35c | G12A | 0.02% |
| KRAS | 1 | 35 | G>T | g35t | G12V | 0.02% |
| KRAS | 1 | 38 | G>A | g38a | G13D | 0.02% |
| KRAS | 2 | 183 | A>C | a183c | Q61H | 0.02% |
| KRAS | 3 | 436 | G>A | g436a | A146T | 0.02% |
| NRAS | 3 | 181 | C>A | c181a | Q61K | 0.02% |
| NRAS | 3 | 182 | A>T | a182t | Q61L | 0.02% |
| PIK3CA | 9 | 1624 | G>A | g1624a | E542K | 0.02% |
| PIK3CA | 20 | 3140 | A>G | a3140g | H1047R | 0.02% |

**Supplementary table 1: List of mutations studied in plasma BEAMing analysis**
