# Supplementary material for: Tumour pharmacodynamics and circulating cell free DNA in patients with refractory colorectal carcinoma treated with regorafenib
Source: J Transl Med. 2015 Feb 12;13:57. doi: 10.1186/s12967-015-0405-4 (PMC4332724; doi:10.1186/s12967-015-0405-4)
Supplement: Additional file 3: Figure S1. — Time course of total plasma cell free DNA. Figure S2. Trend of % mutant cell free DNA with treatment time. [file 12967_2015_405_MOESM3_ESM.pdf]

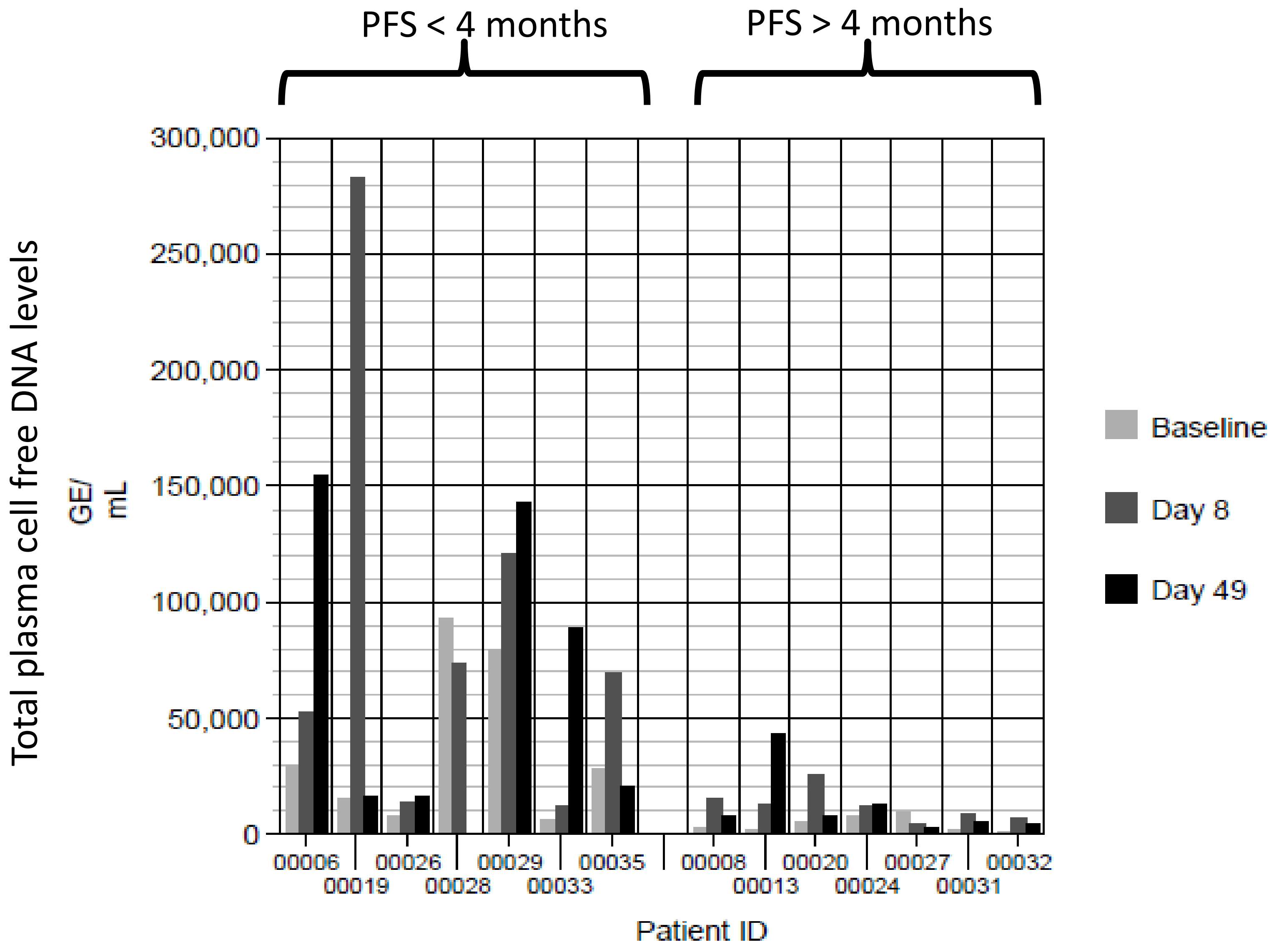

Figure 1 (supplementary) Time course of total plasma cell free DNA

Figure 2A – patients with PFS > 4 months

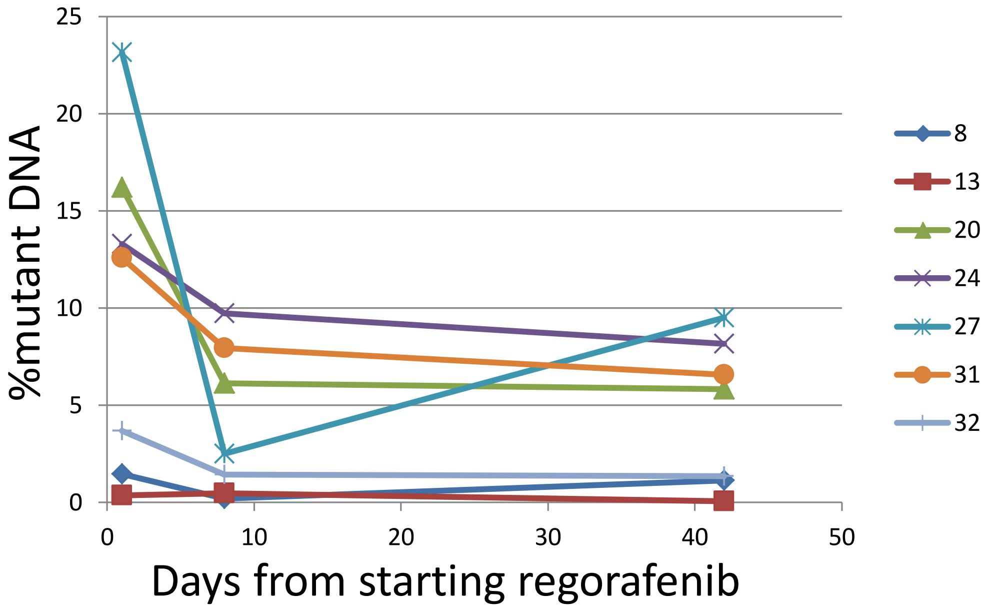

Figure 2B – patient with PFS < 4 months

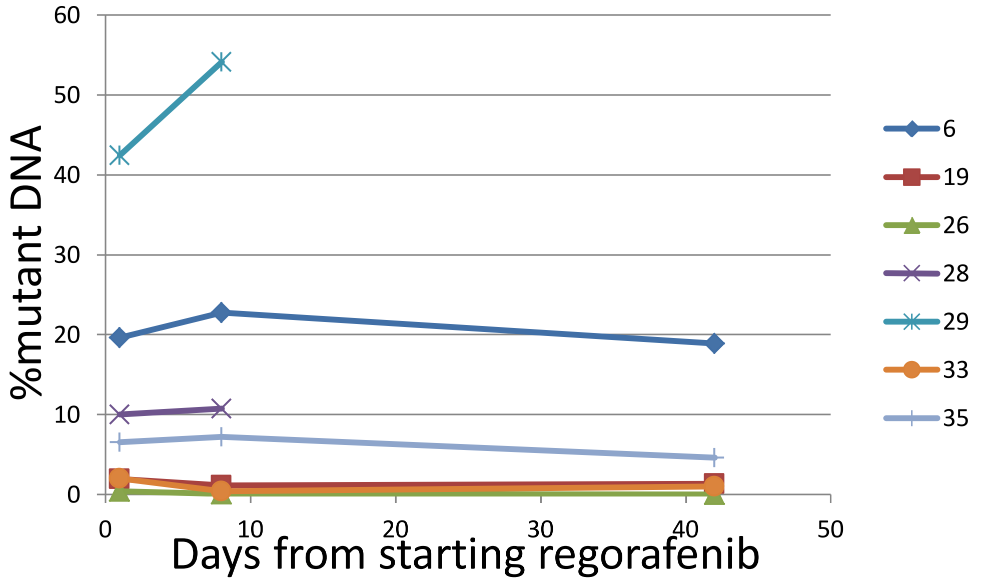

Figure 2(supplementary) – Trend of % mutant cell free DNA with treatment time
